# A qualitative exploration of women’s expectations of birth and knowledge of birth interventions following antenatal education

**DOI:** 10.1186/s12884-024-07066-x

**Published:** 2024-12-28

**Authors:** Anna Davies, Michael Larkin, Lucy Willis, Narendra Mampitiya, Mary Lynch, Miriam Toolan, Abigail Fraser, Kate Rawling, Rachel Plachcinski, Sonia Barnfield, Margaret Smith, Christy Burden, Abi Merriel

**Affiliations:** 1 Academic Women’s Health Unit, Bristol Medical School, University of Bristol, 5 Tyndall Avenue, Bristol, BS8 1UD UK; 2 North Bristol NHS Trust, Southmead Hospital, Southmead Road, Bristol, BS10 5NB UK; 3 College of Health and Life Sciences, Aston University, Birmingham, B4 7ET UK; 4 National Institute for Health Research Bristol Biomedical Research Centre, University Hospitals Bristol and Weston NHS Foundation Trust and University of Bristol, Oakfield House, Oakfield Grove, Bristol, BS8 2BN UK; 5 Public Patient Involvement stakeholder ACE Study, University of Bristol, 5 Tyndall Avenue, Bristol, BS8 1UD UK; 6 National Childbirth Trust, Brunel House, 11 The Promenade, Clifton Down, Bristol, BS8 3NG UK; 7 Department of Women’s and Children’s Health, Institute of Life Course and Medical Sciences, University of Liverpool, Liverpool Women’s Hospital, Crown Street, L8 7SS Liverpool, UK

**Keywords:** Antenatal education, Birth expectations, Psychological wellbeing, Focus groups, Qualitative

## Abstract

**Background:**

Expectations of birth, and whether they are met, influence postnatal psychological wellbeing. Intrapartum interventions, for example induction of labour, are increasing due to a changing pregnant population and evolving evidence, which may contribute to a mismatch between expectations and birth experience. NICE recommends antenatal education (ANE) to prepare women for labour and birth, but there is no mandated UK National Health Service (NHS) ANE curriculum. We aimed to explore women’s expectations of childbirth and their understanding of common interventions and complications following NHS and non-NHS ANE.

**Method:**

Qualitative focus groups were conducted with postnatal women (< 12 months postpartum) aged *≥* 16, who had received antenatal care at a single NHS Trust. A semi-structured topic guide was used to explore birth expectations following attendance at ANE and knowledge of birth interventions and complications. Data were transcribed and thematic analysis was undertaken by at least two researchers.

**Results:**

46 women (mean age: 33.5years; 81% white British) participated across eight groups. 65% were primiparous, 35% had a caesarean birth. 50% attended NHS ANE and 59% non-NHS ANE. Participants perceived that a ‘hierarchy of birth’ was presented within ANE classes, where a ‘better birth’ involved vaginal birth, minimal pain relief and limited intervention. Participants described expectations of control and choice over their birth, though some described being encouraged to be open-minded about the course it may take. Participants identified a mismatch between their expectations and subsequent experiences, which adversely impacted their psychological wellbeing. While participants received information about common birth interventions and complications, limited time spent on these during classes resulted in expectations that they were rare. Participants felt that receiving sensitively presented information about the frequency of interventions could prepare women and support their psychological wellbeing after birth.

**Conclusions:**

Women’s expectations of birth are informed by ANE which may precipitate a mismatch between expectations and experience. Better information about risk factors and frequency of labour and birth interventions may support women to develop evidence-informed expectations of birth, reducing the expectation-experience gap, with consequent impact on maternal postnatal wellbeing. A mandatory minimum curriculum for ANE is needed to ensure high-quality education is available to all.

**Supplementary Information:**

The online version contains supplementary material available at 10.1186/s12884-024-07066-x.

## Introduction

Negative or traumatic birth experiences are associated with postnatal depression, anxiety and post-traumatic stress disorder (PTSD) [1, 2]. Poorer psychological wellbeing in mothers, including perinatal PTSD, is associated with impaired mother-infant attachment [3], which impacts on children’s developmental and psychological outcomes [4, 5]. 

Expectations of birth relating to place, pain management choices and interventions can influence birth experience and satisfaction. There is consistent evidence that a dissonance between expectations and experience is associated with decreased satisfaction and poorer psychological health; in studies exploring impact of a mismatch between women’s birth preferences and actual mode of delivery, women who experienced a birth that was divergent from their birth preferences were more likely to report decreased satisfaction and PTSD [6–8]. Experiencing unanticipated birth complications and/or interventions is associated with negative ratings of birth experience [9], and unplanned caesarean birth and instrumental vaginal birth are associated with increased likelihood of PTSD [10]. In a meta-analysis of 50 studies, risk of postnatal PTSD was associated with birth experience, lack of control and agency, operative birth, lack of support during birth, and dissociation [1]. Conversely, where a birth is consistent with women’s birth preferences improved birth satisfaction is found [11]. 

The UK National Institute of Health and Care Excellence (NICE) recommends antenatal education (ANE) programmes which should include topics such as preparing for labour and birth, and infant and postnatal care, although a detailed curriculum is not provided [12]. Similarly, there are ANE delivery standards in Ireland, but they do not provide detailed content [13]. A systematic review of the effectiveness of ANE identified limited studies of uncertain quality due to poor reporting of methodology within the included studies. Data from included studies indicates potential benefit for increased knowledge for both prospective parents, length of labour, mother-infant attachment, and satisfaction with maternal role preparation [14]. 

Women’s expectations of birth are informed by their prior experiences, the experiences of family and friends, the media, information they access, and their care providers, as well as ANE [15]. To date there has been little exploration of the role of ANE in informing women’s expectations of childbirth [16]. High-quality ANE programmes should inform birth expectations in the context of current clinical care. Factors including increasing maternal age [17], obesity [18], maternal request [19], and changes to national guidelines (e.g. NICE Induction of Labour guidelines [20]) are associated with increased rates of induction of labour and caesarean birth in the UK in recent years [21, 22]. While there are local and regional variations in rates of interventions for labour and birth [23], the rising caesarean rate, reaching 38% of births in 2023 [24], highlights a potential need for women to be informed that different types of birth are a possibility. It is unclear to what extent NHS and privately-provided ANE reflect these practice changes, which may inform women’s childbirth expectations.

### Aims

We aimed to qualitatively explore women’s expectations of birth in relation to their experiences of antenatal education.

## Method

We use the terms ‘woman’ or ‘mother’ throughout. These should be taken to include people who do not identify as women but who are pregnant [25]. 

### Clinical trial registration

Not applicable.

### Patient and public involvement

A patient representative was a member of the study steering group and gave input into the study design, protocol and materials, and is a co-author for this study.

### Design and setting

A qualitative focus group study was undertaken at an acute NHS teaching hospital in South-west England with approximately 5,500 births per year. While both individual interviews and focus groups were considered, focus groups were selected as the means to gather data to address the research question; they enable rapid collection of data and are used to elicit a wide range of views using interaction between the participants to understand, explore and clarify views, taking account of differing viewpoints [26]. The interactions between participants can be used to identify both common and divergent views and experiences of ANE [27], and we believed that the social environment would enable generation of data that may not be provided through individual interviews [28], particularly when talking about emotionally-charged experiences. The study team was multidisciplinary, comprising a psychologist, obstetricians, community and hospital midwives, and parent representatives.

### Recruitment and sampling

Postnatal women were recruited to explore women’s expectations following ANE as well as their experiences of childbirth. Participants were required to meet the following inclusion criteria:

- Age over 16.
- Received their antenatal care at the Trust in the preceding 12 months.
- Able to speak and understand English adequately to participate in a focus group in English.
- Able to provide written, informed consent to participate.

Women were ineligible to take part if they were aged under 16 or could not meaningfully participate in a focus group in English.

Recruitment was between June and August 2019 via social media advertisements on Facebook pages, including NHS Trust maternity services, mother and baby groups, infant feeding groups, local buying and selling pages. Interested participants were offered a convenient date and time to join a focus group in a local NHS hub.

Following the initial recruitment phase, we identified limited participation from women of Black, Asian and minority ethnicity groups, and women without tertiary level education. Consequently, we purposively recruited a group of mothers from a single local authority Children’s Centre for a focus group at the site, to increase the diversity of the sample.

Women were recruited into groups of six to eight to maximise participation, and attended with their babies to facilitate access. Groups were face-to-face and held at hospital sites, and a local Children’s Centre. Women were reimbursed £20 for their expenses.

### Data collection

Participants completed a demographic questionnaire to indicate age, ethnic group, education, parity, gravidity, birth interventions, perceived complicated birth (self-defined), and ANE classes attended.

A topic guide addressed: ANE attended, birth expectations following ANE, and understanding and knowledge of common birth-related interventions. Focus groups were recorded and transcribed verbatim. Prior to use, the topic guide was reviewed by the PPI contributor (KR) for clarity and appropriateness.

### Analysis

**Fig. 1:**
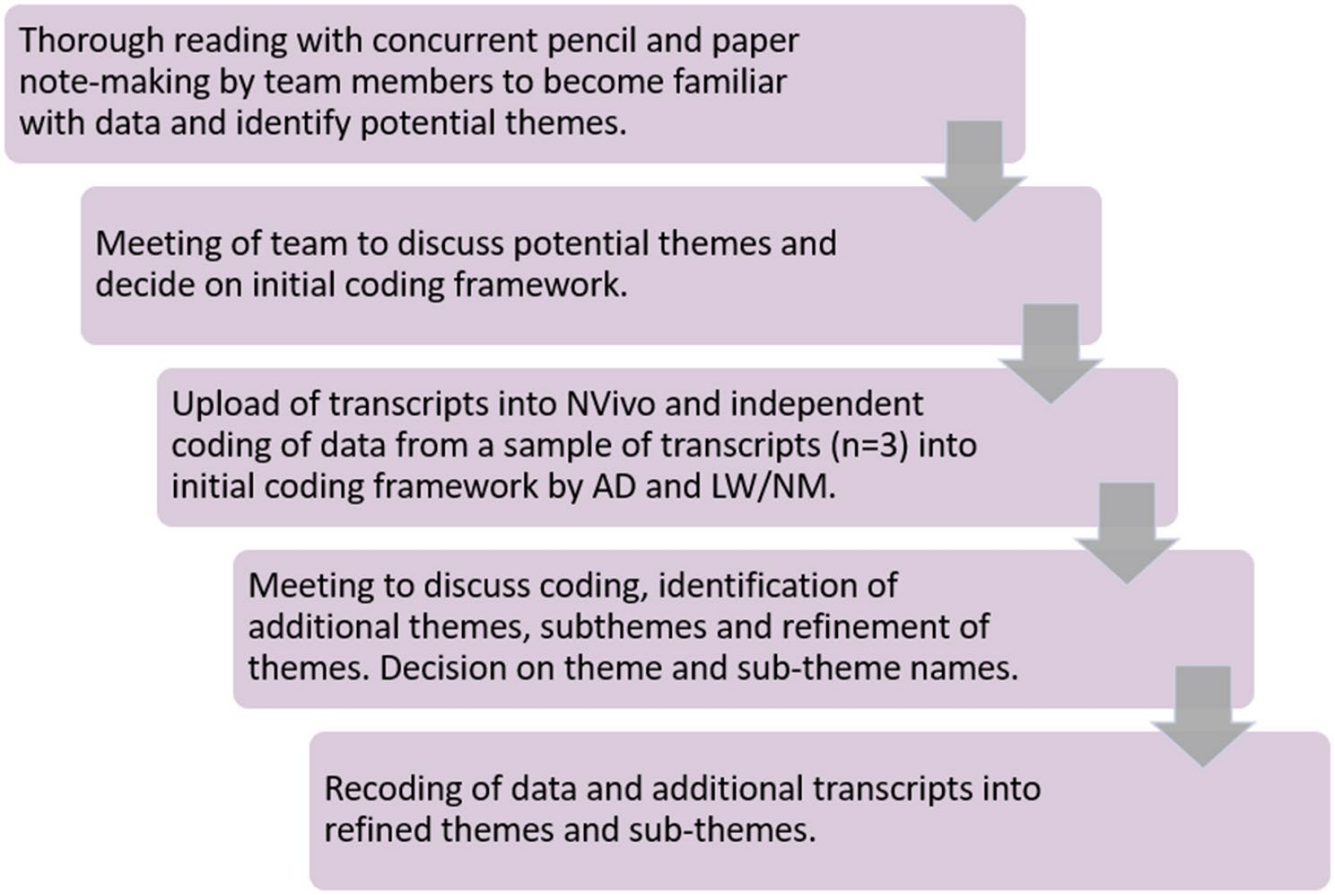
Thematic analysis process

Data collection and analysis were iterative. Potential themes identified in the data from the initial focus groups were discussed by the research team (AD/MLy/MT/AM/ML) and used to refine the topic guide for later groups. The refinement related to perineal tears and episiotomy, which arose in the first focus group, in which the women expressed that they lacked knowledge about this issue and that it was a key concern for them. We therefore explored this in subsequent groups. Transcripts were uploaded to NVivo and analysed in duplicate by AD and LW/ NM to increase validity of coding. Thematic analysis was used to identify themes within the data [29]. An inductive and deductive approach was taken, whereby data relating to specific questions were analysed, with inductive analysis to identify unanticipated themes. The process is described in Fig. 1.

### Reflexive accounting

The lead author (AD) is a health psychology researcher with experience of qualitative research methods and exploring women’s experiences of maternity healthcare. The senior author (AM) is an obstetrician and experienced qualitative researcher. Other researchers included a trainee obstetrician (MT), a research midwife working on delivery suite (MLy) and trainee doctors (NM, LW). To address differences in perspectives between the research team and participants, we ensured that we purposively sampled participants with a range of birth experiences, specifically focusing on *their* perspective of their birth (whether they perceived it to be complicated), rather than our understanding of what is a clinically complicated birth. We iteratively developed the topic guide to ensure that questions could invite a range of differing views about ANE experience. Finally, within the analyses we worked with a senior qualitative researcher who was not routinely involved in maternity research to support analyses and writing (ML).

## Results

Forty-six women participated in seven focus groups. Participants’ demographic characteristics are presented in Table 1. A breakdown of demographic characteristics of each group is provided in supplementary file 1. The majority were aged over 30, white British (80.5%) and had a degree level of education. Sixty-five per cent were primiparous. Over 70% of participants had been invited to attend NHS ANE during one of their pregnancies, and 50% of participants had attended it. 54% had a vaginal birth (with or without induction of labour), 8.7% had an instrumental vaginal birth and 34.9% had a caesarean birth (planned or unplanned). 41% reported having experienced complications (self-defined) during birth.

**Fig. 2:**
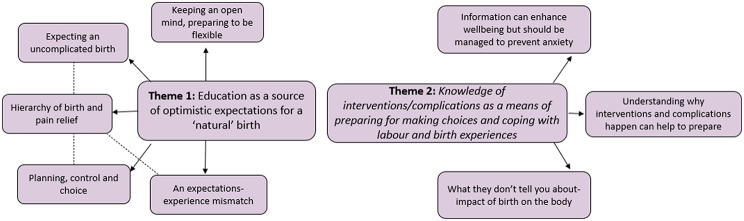
Themes and subthemes

We analysed the data in relation to women’s expectations of birth following ANE, and knowledge and understanding of interventions and risk. Data were organised and constructed into themes and subthemes, which are presented in Fig. 2. We explored patterns both within and across the focus groups and have focused on data where there were consistent themes either across or within groups.

We identified two themes: (1) *Education as a source of optimistic expectations for a ‘natural’ birth* and (2) *Knowledge of interventions/complications as a means of preparing for making choices and coping with labour and birth experiences.*

In the first theme, sub-themes were: (i) *Hierarchy of birth and pain relief*,* (ii) Expecting an uncomplicated birth*,* (iii) Planning*,* control and choice*,* (iv) Keeping an open mind*,* and preparing to be flexible*,* (v) An expectations-experience mismatch.* In the second theme, sub-themes were: (i) *Information can enhance wellbeing but should be managed to prevent anxiety*,* (ii) Understanding why interventions and complications happen can help to prepare*,* (iii) What they don’t tell you about- impact of birth on the body.* Additional quotes representing these themes are provided in Supplementary files 2 and 3.

**Table 1:** Participant demographic characteristics

(*N* = 46)	No. respondents	Total (%)
Age		
*20–30*	9	19.6
*31–40*	34	73.9
*41+*	2	4.3
*Did not indicate*	1	2.2
Ethnicity		0
*White British*	37	80.5
*White: Any other white background*	5	10.9
*Black (British)*,* Asian (British) or other minority ethnicity*	3	4.4
*Did not indicate*	1	2.2
Level of education		
*GCSE/A’-Levels*	4	8.8
*Bachelors Degree/Equivalent*	24	52.2
*Post-graduate Degree*	17	37
*Did not indicate*	1	2.2
Invited to NHS ANE		
*Yes*	36	78.3
*No*	10	21.7
Attended NHS ANE		
*Yes*	23	50.0
*No*	23	50.0
Attended Private ANE		
*Yes*	27	58.7
*No*	19	41.4
Attended both Private and NHS ANE	10	21.8
Age of baby months		
*0–3 months*	8	17.4
*3–6 months*	13	28.3
*6 + months*	25	54.3
First or subsequent baby		
*First*	30	65.3
*Subsequent*	15	32.7
Mode of birth		
*Unassisted vaginal*	26	56.5
*Instrumental*	4	8.7
*C-section: Unplanned(emergency)*	5	10.9
*C-section: Not specified*	7	15.2
*C-section: Planned*	4	8.7
Change in location during birth		
*Yes*	6	13.1
*No*	40	87
Any complications in birth (self-defined)		
*Yes*	19	41.4
*No*	27	58.7

## Theme 1: Education as a source of optimistic expectations for a ‘natural’ birth

### Hierarchy of birth and pain relief

This theme was explicitly articulated in our first focus group, but was further supported by secondary evidence in subsequent groups. In Focus Group 1 (FG1) several women described being presented with a ‘*hierarchy of birth and of pain relief’* within the variety of antenatal classes they had attended. Five had attended a paid-for (private) class, four had attended NHS provision, and three attended both. Women perceived that ‘natural’ births involving minimal intervention and/or pain relief were presented to them by class leaders as being at the top of the hierarchy, with those involving more intervention or pain relief, such as an epidural, placed further down.

Participants identified that in the context of this hierarchy, if a ‘natural’ birth was not achieved, they would feel that they had failed. Notably, participants perceived that medical intervention was ‘bad’, suggesting value judgement around women’s choices about, or identified clinical need to have, interventions to support them in labour and birth. One participant reflected that having a baby without pain relief or complication was not ‘*achieving something’.* Participants explicitly acknowledged the potential relationship between expectations and experiences in this context:

*There was a clear hierarchy. For me*,* my preference was to have a more natural birth and that that depended on what happened. I think if you weren’t that pragmatic you could have quite a traumatic experience if it didn’t go that way for you. (FG1)*

Participants discussed how this hierarchy could impact on mental wellbeing, leaving them feeling negative about their birth. One participant contrasted this narrative with other clinical situations in which pain was experienced and medical care was offered, where it would be unusual to refuse pain relief. The need for a more neutral discussion of options for pain relief and differing births was discussed by participants, to reassure women that whatever course their labour and birth took, they could still feel good about it.

*I just don’t think there’s really many other things in life*,* especially medical things that cause pain that you would be like of course I don’t want painkillers but you’re sawing off my leg or something. It’s got some sort of specialness about it*,* that your body knows and all of this. It’s like*,* I don’t know*,* I think it then relates to people feeling more bad about it. (FG1).*

The hierarchy presented to participants implied a ‘correct’ or ‘best’ way to labour and give birth, and this narrative was promoted in both the NHS and private classes. This ideal birth did not reflect consideration and promotion of individual choice and care, in which the safest, most comfortable birth for the individual mother and her baby was advocated. Instead, an unnuanced approach promoted birth where pain relief medication or intervention are avoided as being more ‘natural’ and having a ‘specialness’.

### Expecting an uncomplicated birth

Although the perceived *hierarchy of birth and pain relief* was not explicitly discussed in other groups, its impact was apparent in the expectations described in response to ANE. When asked about their birth expectations following ANE, participants across focus groups reported that they expected an uncomplicated, ‘natural’ birth, anticipating limited medical interventions to assist delivery or pain relief.

They linked this expectation to the emphasis and time given to discussing interventions; in one focus group, a participant felt her expectations were informed by the facilitator conveying that complications or interventions were unlikely or exceptional, and therefore unnecessary to learn about:

*They never actually said ‘you won’t need to know’*,* to be fair to them*,* but it was done in such a way that it would be like ‘well*,* this is the exception’ kind of way and actually looking at it now it’s not! (FG6)*

Similarly, participants across groups recognised that the emphasis of the classes attended had been on having a birth that did not involve medical intervention:

*Yes the [name] classes*,* I remember focussing so much on how sort of the lovely ways that you could give birth. …. Well yes*,* they don’t prepare you enough for actually you’re gonna walk into a room*,* you’re gonna have an amazing midwife who’s gonna press a button if your baby poos when it’s coming out and you’re gonna have a team of doctors. That won’t happen in a field. [laugh] (FG3)*.

*I think I had a very difficult labour*,* I didn’t know it could be [inaudible − 15:35]*,* because it’s like the*,* like you said*,* the perfect scenario*,* they’re not just trying to scare you*,* but the other options that were available. So if you get pre-eclampsia you might have to stay in for a week ’cause …. These are things that might count as an emergency…. it’s almost just like there could never be anything wrong (FG7)*.

In relation to pain, a participant described how, in the context of a low intervention, physiological labour and birth, she perceived a pressure for women to enjoy their birth, reflecting that even with a straightforward physiological birth, there was potential for pain and trauma, which she felt women were underinformed about. For example:

*Yeah*,* I don’t think it was realistic. The [private provider] ones for me just felt like you should really enjoy it because it’s natural*,* because we’re animals and we can do it*,* it’s going to be a lovely experience and I think that’s really naïve because even if you have a textbook labour everyone I know says it hurts and it’s really traumatic so it would have helped to have been told that! (FG1)*

While participants considered it important to avoid making women anxious, they felt that avoiding discussion of interventions could affect the wellbeing of women who do not experience this ideal birth. Participants described feeling they had been misled, or ‘kept in the dark’ by a lack of information about complications.

I think what’s interesting about just looking at something like this is, I think with the whole positive birth movement and only talking about things kind of naturally has been such a good thing, but I think it also the way, the way it filters down to like the midwife that you see on your, you know, normal routine visits before you give birth, it’s almost like rather than being empowering, it’s keeping women in the dark about their own bodies and about the process which you’re going to go actually go through (FG2).

One participant described how this made her feel she had ‘not given birth’:

Yeah and then you might just feel – I just feel like it didn’t … birth but, similar to you, I felt like I hadn’t given birth and I think if it had been covered a bit more, it’s just a type of birth and yeah, so yeah. (FG7)

The balance and focus of information also fostered expectations about place of birth, with some perceiving that they had been given unrealistic expectations about birthing in a midwife-led environment, again linked to the ideal ‘natural’ physiological birth. They reflected that this could impact women’s wellbeing, *‘setting them up for a fall’* (FG1) or feeling like they had *‘failed’* (FG2) if they missed out on this experience. This was reflected by another participant also:

The amount of time and focus within ANE on a physiological birth in a low intervention setting, and limited attention given to birth interventions or birth in a labour ward led women in this study to believe that this was the most likely birth experience. ANE was perceived by women across focus groups to be unbalanced, misleading, with the potential to harm their wellbeing in the event that this ideal birth and setting was not achieved. Even when the ideal birth was experienced, the additional expectation that they should enjoy labour and birth added to potentially unrealistic expectations. Unmanageable pain during childbirth is not a universal experience, however, women felt again that their birth may not meet the acceptable standard if they did not enjoy it. While participants reflected that it was important to manage women’s fears about labour and birth, they desired realistic information about what birth could be like and the likely location of their birth within their clinical context and current NHS resource.

### Planning, control and choice

The importance of control and choice was a theme evident across groups.

However, some participants perceived that ANE had fostered an expectation that they could control both the mode of birth and whether they experienced pain, giving them *‘false confidence’*. In one group, women reflected that privately provided classes emphasised writing a birth plan, and that the planning terminology used could create an erroneous expectation that women could always exert control over their experience. One participant described how she had not anticipated control over her birth prior to attending ANE, and that it was ‘up to your body what happens’, but reported being surprised that the class she attended was promoting the idea that women could exert control. Another participant from the same group who had attended hypnobirthing training described that an unanticipated loss of control during transition resulted in a sense of catastrophe or loss about not having the ’ideal’ birth experience.

*Especially from the hypnobirthing course that I did first time. It was very much I came away thinking that I could do this. I could control this. I could get through it and control the pain and I was totally and utterly floored*,* how that just for me*,* wasn’t – it did happen up to a point*,* but once I was sort of transition time and then after that I was so*,* so out of control the first time and actually*,* I suppose I might have benefited more from a bit more of a realistic reminding that things can happen really differently and that’s okay too. I think that made me panic. I thought*,* oh everything’s gone*,* everything’s lost.(FG1)*.

In contrast, another participant described a shift in her feelings of perceived control, contrasting an NHS class where she left feeling that she would need pain relief, with a hypnobirthing class, where she felt more empowered.

*With my first after doing the NHS ones*,* I was just like - all the drugs. [laugh] That was pretty much what I came away with was*,* yes. Whereas after doing hypnobirthing*,* it was very different. I felt a lot more positive about it*,* I felt more empowered. (FG3)*

Choice was also discussed in relation to whether choice really existed when asked to consider intervention by the clinical team. One participant described feeling that she had been led to believe during ANE that induction of labour was a choice, but when offered it she felt there was no real choice to make, due to what she perceived was the right course of action for her baby’s safety.

*And I think the [Private Provider] ones very much gave me the impression that it was all my choice and that induction was completely a choice and it probably wouldn’t happen and then I think you can feel like you’ve failed*,* when actually I felt like I had absolutely no choice at all when I was induced and it was definitely the right thing to do for the baby.(FG6)*.

Here we see that ANE frequently led women in this study to perceive birth as a situation in which they are able to plan, exert control and make choices both prior to and during birth. Participating women perceived this to conflict with their experiences, identifying that they could not control how their birth might go, and when faced with a higher-risk birth or a situation they had not anticipated, they felt that their choices were no longer attainable. With hindsight, women wanted ANE to better prepare them for a broader range of eventualities.

### Keeping an open mind, and preparing to be flexible

Importantly, expectations of a low intervention, physiological birth during which control could be exerted were not universal. Several women across all groups discussed feeling ‘*open-minded’* after ANE, hoping only for a healthy baby and believing it wasn’t worthwhile to plan.

*I didn’t have a plan ’cause I was like he’s got to come out*,* there’s no point planning*,* he’s got to come out*,* I understand that it’s not going to be pain free*,* I understand that it’s gonna take a long time. (FG7)*

Some reported that ANE had specifically prepared them for an unpredictable labour, with some attending classes that emphasised hoping for the best, but ensuring they anticipated the unexpected. One participant described being prepared for intervention during her class:

*We were sort of encouraged to hope that everything would go straightforward but we went through a role play of what happens when you go to theatre and what different complications might happen and like why you might want an epidural*,* why*,* why an intervention might happen. (FG4)*

This acknowledgement of potential for intervention increased opportunities to plan for an experience aligned with some of the identified benefits of a physiological birth in the event of complications, such as how to achieve skin-to-skin contact with their baby following caesarean. Women also described how being focused on delivering a healthy baby as the ultimate goal was protective of wellbeing, as it allowed for psychological preparation and prevented disappointment when intervention occurred. For example:

*… my view was whatever needed to happen so yes I’ll go in and I’ll be prepped for a C-section if I need*,* whatever*,* as long as he’s alright I don’t mind*,* so that my birth plan was almost whatever needs to happen. (FG5)*

Notably, however, one participant discussed that there were limits to her open-mindedness, describing how she had retained some preferences relating to avoiding epidural. This resulted in disappointment when this expectation was not met:

*I deliberately had mine [preferences] really open*,* I didn’t want to make any decisions so that I wouldn’t be disappointed but the only thing I didn’t want was an epidural and I ended up with an epidural and then a c-section. So actually at the end of it because I wasn’t prepared for that choice I felt like I’d done a really bad job so I felt quite disappointed myself.(FG4)*.

Here we see that ANE supported some women to develop expectations that prioritised giving birth to a healthy baby, and psychological wellbeing where intervention was encountered. Women in this study valued being encouraged to aim for a straightforward vaginal birth, while preparing for other possibilities, enabling them to consider alternative means to achieve their birth preferences.

### An expectations-experience mismatch

As a consequence of having expectations of a ‘natural’ physiological birth and control over the birth experience discussed in the preceding themes, several participants identified a mismatch between their expectations and experiences. One woman described how she felt well prepared for a birth that was going to go to plan, but unprepared when it did not go as anticipated. Another described how she should have gone to the *‘Oops it all went wrong’* class (FG4). Unexpected pain, interventions and a longer or shorter labour than expected precipitated this mismatch, with one participant not understanding that her birth could be longer than she expected:

*I thought I was gonna go in, have a baby and come out within two hours. (FG7)*

Conversely, one participant reported that her perceptions about the timeframes involved and the potential to be turned away resulted in her not attending hospital as early as she should have:

*I think I expected it to be long and slow which mine really wasn’t. Yeah. I think everyone kind of like very much of the opinion that first babies are very drawn out*,* so I think in my head I was expecting to camp and to be very much I almost put off going to hospital because I was worried about being sent home and actually I needed to see a hospital. (FG1)*

One participant described how, due to having an uncomplicated pregnancy, she had expected the birth to be similarly straightforward, emphasising the experienced mismatch:

*I’d just add that there was a mismatch perhaps with the fact that he was like… I had a textbook pregnancy*,* or so I was constantly told*,* and he was always on the 50th percentile measurements and [….] so I was kind of reassured throughout the pregnancy and then I felt not prepared for what happened.(FG6)*.

Here we see that when women in this study developed expectations about birth there was potential for a mismatch between these expectations and their actual labour and birth. This gap is present when women experience a labour that follows a different path than they expected.

## Theme 2: Knowing about interventions and complications can support collaborative choosing, coping and recovering

We explored the extent to which participants had been informed about interventions used to start or augment labour and to assist birth (e.g. induction, instrumental and caesarean births), and understanding of why they were offered. This provided an opportunity to discuss how they might feel about being told during ANE about interventions and the likelihood of them being offered or recommended. Themes related to the need to balance providing information to support preparedness with a need to avoid precipitating anxiety, how understanding frequency of complications can help prepare for birth, and what information they want but aren’t told.

### Information can enhance wellbeing but should be managed to prevent anxiety

Across groups participants reported that a range of interventions were discussed in ANE at greater or lesser depth, including induction of labour, caesarean birth, and assisted vaginal birth (forceps and vacuum; see supplementary file 3). They perceived that receiving sensitively presented, factual information about birth interventions and complications was important, and could enable decision-making about their birth preferences, including place of birth, for example, to ensure they understood the likelihood that they might need to move from a midwife-led unit to a labour ward setting to access increased medical support.

Women indicated that having factual information about the likelihood of interventions and complications could help them with decisions about place of birth:

*I think it might be useful in thinking about where you’re going to deliver. I suppose if [midwife-led unit]*,* I think knowing all the things that could happen that would mean you need medical assistance*,* having to travel across [city] but if you had the actual statistics as well that would be useful and thinking about that. (FG2)*

One participant felt that having information about interventions could increase fear for women who were already nervous about birth, however, this participant also felt that it was important for women to be realistic about what could happen:

*I think if you’re nervous I think it could be quite scary*,* but I think you have to be realistic*,* ultimately the baby has got to come out so you might as well have all the information*,* rather than this rosy*,* oh*,* it’s gonna go in*,* you’re gonna be coming out in 16 h [inaudible] and then it’s gonna come out in two hours and they pat you on the head and send you home. It’s just not – you have to be realistic. (FG7)*

Participants discussed how having information about interventions and complications could support psychological wellbeing, facilitating acceptance when things had not gone as they had hoped. Similarly, participants recognised that in presenting data about likelihood, it could highlight that many do indeed have a spontaneous vaginal birth, but that there are options when the birth does not go according to plan. For example:

*I mean*,* this*,* this tells you that most births are spontaneous vaginal deliveries. Now*,* for me looking at that*,* I’m like*,* wow*,* that’s amazing. And actually*,* if you*,* if you take the 10% of elective caesareans out of that too*,* it’s even more of a percentage that actually it just works. And*,* but if it doesn’t work*,* these are the things we’re going to do. These are the decisions you might have to make um*,* and actually it’s a very matter of fact thing.(FG2)*.

In one focus group, women highlighted that it could prevent them from feeling that they had done something wrong:

*It would make me more accepting of the outcome of what happened. I don’t know if it would necessarily change*,* because again I don’t think you can decide really. I think you’d have your baby however it happens but your attitude to it afterwards can be really affected by what you know in advance and the likelihood of the chances and that the people that have certain birth experiences haven’t done anything right or wrong. That’s just how their body works.(FG2)*.

However, participants in this group also acknowledged different information preferences. Some preferred to gather as much information as possible, whereas others felt they could be overwhelmed. Optional access to detailed information was discussed, with one participant suggesting that basic information such as statistical likelihood was important, but that it could be useful to allow women to access further information if desired by providing it in a supplementary format, such as factsheet. They felt that it should be provided in ANE classes, however, one participant described being happy to go away and seek information for herself if she wanted to find out more.

*Or having factsheets at the front that you can go and pick up if you want because if you’re going away and finding a website*,* you have to remember and you’ve got loads going on and you’re working. For me having it available in the class. I don’t know if I’m just lazy would be really useful but not flashing it up on a screen so everyone has to look at it. (FG2)*

Here we see that participants largely considered information to be useful in supporting decision-making about place of birth, as well as supporting psychological wellbeing if a birth does not go as anticipated. However, it is important to explore acceptable and potentially tailored ways in which to deliver this information, to meet information preferences and avoid precipitating anxiety.

### Understanding why interventions and complications happen can help to prepare

Across groups women demonstrated knowledge of the reasons why interventions are offered. However, the extent to which their understanding had arisen from ANE or direct experiences of needing intervention was unclear, with some recalling the reasons they had been offered induction of labour or other intervention. Some participants across groups felt that it would be useful to discuss the reasons why particular interventions might be offered to help them prepare for when birth did not go as anticipated.

*It might be good to go through the reasons why people have these types of birth and why people have the natural ones so that the [inaudible] prepared. (FG5)*

Reasons for induction of labour were well understood across groups, with some expressing understanding of recent changes to NICE guidelines resulting in an increased induction rate. Similarly, participants perceived that likelihood varied according to both pregnancy and fetal characteristics, such as baby’s weight, position and size, as well as maternal factors including age and parity. One participant also reflected that if risk factors specifically applied to them, such as family history, they were more likely to be aware of potentially relevant interventions. She related this to older mothers tending to be more engaged with information relevant to them.

*I think also older women tend to be more educated*,* more middle class*,* that is just the trend isn’t it. Those women are having babies later so I think that by and large they’re quite well informed of those risks or they choose to inform themselves at the time quite well*,* but maybe there’s the other section of much younger women. I don’t know. In different kind of areas*,* different. (FG2)*

In this theme we see that participants felt informed about some of the factors that could increase the likelihood of being offered labour and birth interventions, and in particular they were well informed about induction of labour. Notably, they felt well informed if they had specific risk factors such as maternal age, perhaps because it had been discussed with their clinical team, or if there was family history of an assisted delivery. It is unclear the extent to which likelihood of intervention is discussed with those who do not have identified risk for intervention, who may nonetheless experience a labour and birth intervention. Participants recognised that understanding why an intervention may be offered could help to prepare for when birth did not go as expected.

### What they don’t tell you about- impact of birth on the body

Participants also highlighted areas where they wanted information but the information was limited within ANE. A key issue highlighted across groups was recovery from the birth. Several participants felt underinformed about perineal tears, with some reporting that they had been not discussed at all, particularly in NHS classes, despite their frequency. However, discussion of tears varied according to which class they had attended, with more information being offered in the non-NHS setting, and varying information about what was better, with one participant describing that she perceived from her privately provided class that episiotomy was ‘*not the best course’*(FG5).

Women wanted information about how to avoid tears and to be told this before they were in labour:

I think it would be good to have something on how to reduce your risk of tears because I think like sometimes they talk you through pushing when you’re fully in labour but actually to find out how to push and how to deliver the head before you have the baby and before you’re in loads of pain would be a good idea. (FG4)

Additionally, one participant described how tears and episiotomy were a key topic of interest to women in her class, and while she was interested in how to avoid them, she felt it would be useful to know about how to self-care after a tear or episiotomy, because she knew they were common and therefore not necessarily avoidable. Not knowing that she may need to go to theatre to repair a tear was also discussed by one participant (see Supplementary File 3).

*That was like our number one question when they were like what do you want to know about? We want to know how to not tear and I think it would have been useful to have a bit more how to deal with tears once they have happened*,* less how to prevent them happening in the first place because I think they’re quite common and I think everyone was very much*,* oh we want to prevent them*,* maybe a bit more maybe this is what happens.(FG1)*.

Participants also identified that what could happen after the birth of their baby was infrequently discussed, including stitches, support to deliver the placenta, bleeding and breastfeeding. The timeline for recovery and self-care were situations for which participants felt underprepared, with one reporting her recovery to be more difficult than her birth. As described in earlier themes, participants felt that knowing about these issues would have better prepared them for the immediate postnatal phase.

*My friend told me that you bleed for weeks and weeks afterwards. It wasn’t a medical professional. (FG3)*

*I just felt like it yeah*,* delivering placenta*,* having stitches*,* breastfeeding*,* the whole shebang*,* I felt that like I wasn’t expecting this. (FG2)*

*Yeah*,* and I was told a lot because I was young I would heal brilliantly and perfectly but actually I found it a lot harder than the actual giving birth part. (FG6)*

In this sub-theme we see that postnatal women identify information that they believe would have been useful to understand, particularly around the bodily impact of birth. Focus on the birth itself, with minimal attention given to even the immediate post-natal period leaves them underprepared for what to expect, and how to self-care during the physical recovery from birth.

## Discussion

Within this investigation of expectations of labour and birth following ANE, two themes were constructed: (i) *Education as a source of optimistic expectations for a ‘natural’ birth* and (ii) *Knowledge of interventions/complications as a means of preparing for making choices and coping with labour and birth experiences.* Within these themes we identified variation in ANE experiences, and that women derive a set of expectations of labour and birth from ANE.

Some inferred from ANE that labour and birth interventions were uncommon and not a desirable outcome, and some perceived that they could control their labour and birth. Others described being encouraged to be more cautious, preparing themselves for other eventualities than the ideal ‘natural’ physiological birth. Women linked unmet labour and birth expectations to potential for poorer postnatal psychological wellbeing. Participants felt that it was important, and possible, to prepare women for different potential experiences without provoking anxiety or disempowering them.

Women’s accounts of their birth expectations highlighted a pervasive ‘hierarchy of birth’ in ANE, involving a spontaneous labour and a physiological birth, which minimised or avoided pain relief, intervention and episiotomy at the pinnacle. This implicit hierarchy appears to have informed the education of many participants; it impacted the amount of focus and time given to informing women about the range of birth experiences they might expect. This was inferred by women to mean that they need not expect intervention. Participants also reported that the amount of time afforded to interventions in private provision was greater, indicating unequal access to information about childbirth for those who cannot access privately provided ANE. Consequently, many participants felt unprepared for their birth when interventions or complications were experienced, even where interventions were common, and reported that unmet expectations of labour and birth resulting from this pervasive hierarchy impacts their wellbeing. If expectations are met a positive birth experience is likely. However, within our data, women perceived that not experiencing this ideal birth could adversely impact mental health, and lead to feelings of failure, disappointment, and self-blame. Our findings are commensurate with those of previous studies exploring correlates of psychological morbidity, in which unexpected intervention such as unplanned caesarean birth is associated with increased likelihood of PTSD and decreased birth satisfaction [1]. 

Conversely, some women reported good practice in ANE. They felt encouraged to remain open-minded about what may happen. As a result they developed plans that could make their birth experience more like a ‘natural’ physiological birth if desired, with the aim of preventing disappointment and supporting psychological wellbeing if complications or intervention were encountered.

Our data support the assertion that women should be encouraged to develop expectations that are well-aligned with current clinical care and experience; recent data suggests that the majority of births involve some form of intervention even where birth is vaginal and uncomplicated; for example, in a study of vaginal births, one third of women had experienced induction or augmentation of labour [30]. Censorship of information about what may happen during labour and birth, referred to by participants as ‘pulling the wool over our eyes’ and ‘keeping women in the dark about their own bodies’ can be considered a form of epistemic injustice; [31] omission of information about the range of potential labour and birth experiences is misleading women to infer that births typically follow this ideal course, and has the potential to cause psychological harm.

This perceived hierarchy is likely to stem from current concerns within the clinical and wider community about the medicalisation of pregnancy and birth, and evidence of better clinical outcomes associated with physiological birth [32, 33]. It may also relate to who delivers ANE in both the NHS and private settings; in our setting providers are typically Community Midwives, whose role is intrinsically linked to supporting physiological birth, and many of whom may not be directly involved in intrapartum care. Similarly, non-NHS providers may have limited direct experience of current clinical practices. A survey of maternity staff and women in Australia has indicated that staff overestimate the likelihood of an uncomplicated labour and birth without sutures in comparison with current local data [34]. 

ANE informed many participants’ expectations about control and choice during their labour and birth. This conflicted with their experiences, when they could not give birth in their chosen setting or felt that limited options were available to them, due to potential impact on their baby’s safety. This may have resulted from how risk was communicated with women, resulting in them feeling less choice around decisions than there was in reality. In a study exploring the views of professionals around shared decision-making, clinicians reported using both their clinical experience and training, but also their preferences, as to how information was presented to women, resulting in what they described as ‘clinical or personal bias’ [35]. Presenting unbiased information about choices could support women to have a greater sense of control and choice about decision making. In the current study, some participants reported feelings of loss of control when their birth was more difficult or painful than expected, including during transition. Notably, feelings of loss of control are commonly experienced during physiological birth, and women should be well-informed about this. Conversely, some women had been encouraged during ANE to remain open-minded, and to prepare for a range of eventualities, referring to birth preferences rather than plans. They reported focusing on the baby’s health as the most important outcome of their birth, attributing these strategies to their ability to cope with a birth involving intervention. This finding is supported by a systematic review of the qualitative literature, in which women report wanting to retain a sense of control and of achievement even when birth has not gone as anticipated [36]. 

A sense of control and agency during labour and birth have been linked with improved birth satisfaction and psychosocial outcomes [37]. This is recognised within NHS care frameworks, and enabling women to express their choices during labour and birth is a vital element of the care that should be offered [38]. However, control may mean different things to different women, including control over decision-making for some, and feeling safe to relinquish decision-making to healthcare practitioners to others [39]. ANE may be failing to provide women with an understanding of how they can express agency. It may be beneficial to frame women’s expectations of control over their birth and decision-making in terms of preparation for a dynamic situation. They should be aware of the range of choices they may need to make, the opportunities that they will have to exert agency, and that different choices may arise in response to previous care, choices, and in the context of the developing clinical picture. Positively framing these choices in terms of making them with the support of midwives and doctors for the benefit of their baby may improve psychosocial outcomes where difficult choices need to be made. For example, there is increasing opportunity to have a ‘natural’ caesarean birth, to improve women’s experience [40]. It is important to note that women are frequently asked to make decisions about care and interventions during their labour and birth.

We did not explore participants’ perceptions of whether offered interventions are justified or women’s experiences of decision-making with clinicians, and this topic did not arise within the focus group discussions. How to prepare women during ANE to discuss and make informed decisions around recommended interventions with their clinical team could be explored in future research. Systematic reviews of quantitative [41] and qualitative data [42] relating to Continuity of Care (CoC models) suggest that women are more satisfied with their experiences and feel more personalised care, empowerment and involvement in decision-making when cared for in a CoC model compared with other care models [42]. Quantitative data indicate that women cared for in a CoC model compared with other models of care are more likely to experience vaginal birth and less likely to experience regional analgesia, instrumental birth and pre-term birth (birth < 37 weeks gestation) and have a probable reduction in fetal loss [41]. This suggests that CoC models could support greater personalisation of care for labour and birth, enabling women to receive information that is tailored to their needs, to increase empowerment and involvement in decision making, and could reduce the need for interventions during birth. Midwife-led CoC models of care may also provide an opportunity to support women to feel more empowered around decision-making and reduce the expectations-experience gap.

We identified that women in this study were aware of interventions and complications, and had good understanding of the reasons for them, although it was unclear whether this understanding was derived from ANE or from their birth experiences. Women in this study reported limited understanding of detail relating to interventions, their frequency and how they might relate to their own pregnancy if they did not have specific risk factors for them. Women reported that they would find information about rates of intervention and reasons for them helpful in normalising their birth experiences, believing that it could prevent potential negative wellbeing impacts if more intervention than desired was experienced. It is reasonable for NHS maternity services to be able to report this data to women to provide information about rates of intervention for labour and birth in their maternity unit.

Women recognised individuals’ differing informational needs, but acknowledged the importance of receiving information about common birth experiences and complications, highlighting that while it may make some women anxious, it was important to consider *how* to deliver this information, not whether it should be offered at all. One notable finding related to perineal tears; women reported receiving limited information about them and recognised that they needed to know what to do about them, rather than how to avoid them, since some degree of perineal injury is estimated to occur in 9 in 10 first-time vaginal births [43, 44]. It is possible that avoidance of providing information about the frequency of non-physiological births and common complications such as blood loss or perineal tears is due to ANE educators’ concerns that it will provoke anxiety, or disempower women around having a physiological birth. However, we have not identified any evidence to support this assertion. Furthermore, we have not identified any evidence to suggest that women without antenatal indications or fear of childbirth are less likely to opt for a physiological birth if they are informed about the likelihood of, and risk factors for, birth intervention or common complications.

### Implications for practice and research

ANE, particularly where provided by the NHS, should be accurate and grounded in current clinical practices, ideally incorporating information and data about local care practices and rates of interventions, to increase women’s opportunity to have a positive birth experience that meets expectations. Women should be supported to understand available choices and when control can be exerted in relation to their own preferences, but that there is a need to frame expectations in relation to how they can operate within a dynamic situation. Further research in women attending ANE could explore how to deliver information about interventions while minimising fear of childbirth.

Better-quality ANE is needed for women attending both NHS and non-NHS provision, informed by data about what women want and what maternity staff think is important to understand. Women and providers working together to co-produce ANE content will ensure it delivers information considered to be important by both parties, and we have successfully co-produced a labour and birth ANE session in this way [45]. Co-production methodology has also been used elsewhere in maternity services in a project aiming to increase shared decision making [46]. A minimum curriculum for ANE and training for staff should be established and advised by the Royal Colleges or NICE, to support standardisation of this aspect of care.

### Strengths and limitations

This study explored the experiences of a large group of women who had attended both NHS and privately provided ANE and had a wide range of birth experiences commensurate with local data, though it is notable that over 40% of them perceived they had had a complicated birth.

A multidisciplinary team worked together to code the data into themes, increasing the validity of the findings. Nonetheless, it is acknowledged that in gathering data from postnatal women, their recall of ANE was explored and may be impacted by their birth experiences. In future, it may be beneficial to explore women’s experiences immediately after ANE and to follow them up soon after birth to explore the impact of ANE on the concordance between expectations and experience.

It is also possible that a self-selecting group of women came forward, who had particular experiences or views of ANE, and the extent to which these experiences generalise more widely within and beyond a single NHS Trust should be explored.

We did not transcribe the focus groups to enable individual speakers to be identifiable. This meant we were unable to connect the pregnancy and birth experiences or the types of ANE individuals had attended to their experiences of ANE and the expectations-experience relationship. Individual interviews could facilitate greater exploration of this issue.

We explored the experiences of women who had attended a wide variety of ANE, including NHS classes, private antenatal preparation classes and classes specific to hypnobirthing, with the aim of exploring ANE experiences more broadly. In future it may be useful to explore in greater depth experiences of specific classes, to compare them, since they can be perceived to have differing purposes.

We experienced difficulty in recruiting women from underserved groups, including those from Black, Asian and other minority ethnicities, and women from less educated and lower socioeconomic status backgrounds. Similarly, this study was not resourced to conduct focus groups in women who do not speak English as a first language. Given identified disparities in pregnancy outcomes in these groups, further exploration of their ANE experiences is important to ensure their representation when developing future ANE. Difficulties in recruiting women from underserved groups and barriers to their recruitment have been previously described [47]. Our attempts to achieve diverse recruitment via social media and the NHS were unsuccessful. This may have related to the approach used. We addressed this by undertaking a focus group in a local authority Children’s Centre with a mother and baby group attended by women from minority ethnicity groups who had established relationships with one another. It is also possible that individual interviews could be more appealing to these women as a means to gather data.

Finally, the views of fathers and non-birthing partners were not sought and should be explored in future research to understand their birth expectations and experiences of ANE.

## Conclusion

This study explored expectations and understanding of interventions following ANE. Women reported that ANE informed their expectations, which they perceived to be influenced by a focus on physiological birth with limited use of pain relief. These expectations can result in a mismatch with birth experiences, with potential for detrimental effects on psychological wellbeing. ANE is also precipitating expectations of control and choice that are not met. Providing information that is presented neutrally, consistently and supportively by NHS and non-NHS providers could enable all women to have birth experiences that are more closely mapped to their expectations.

## Electronic Supplementary Material

Below is the link to the electronic supplementary material.

Supplementary Material 1

Supplementary Material 2

Supplementary Material 3

## Data Availability

The dataset used and analysed within the current article is not publicly available, however can be made available on request, with appropriate ethical permissions in place.

## Abbreviations

ANE: Antenatal education
PTSD: Post-traumatic stress disorder
NHS: National Health Service (UK)
NICE: (UK) National Institute of Health and Care Excellence

## References

[1] Ayers S, Bond R, Bertullies S, Wijma K. The aetiology of post-traumatic stress following childbirth: a meta-analysis and theoretical framework. Psychol Med. 2016;46(6):1121–34. 26878223 10.1017/S0033291715002706

[2] Furuta M, Sandall J, Cooper D, Bick D. The relationship between severe maternal morbidity and psychological health symptoms at 6–8 weeks postpartum: a prospective cohort study in one English maternity unit. BMC Pregnancy Childbirth. 2014;14(1):133. 24708797 10.1186/1471-2393-14-133 PMC4021064

[3] Ayers S, Wright DB, Wells N. Symptoms of post-traumatic stress disorder in couples after birth: association with the couple’s relationship and parent–baby bond. J Reproductive Infant Psychol. 2007;25(1):40–50.

[4] Parfitt YM, Ayers S. The effect of post-natal symptoms of post‐traumatic stress and depression on the couple’s relationship and parent–baby bond. J Reproductive Infant Psychol. 2009;27(2):127–42.

[5] Cook N, Ayers S, Horsch A. Maternal posttraumatic stress disorder during the perinatal period and child outcomes: A systematic review. J Affect Disord. 2018;225:18–31. 28777972 10.1016/j.jad.2017.07.045

[6] Webb R, Ayers S, Bogaerts A, Jeličić L, Pawlicka P, Van Haeken S, et al. When birth is not as expected: a systematic review of the impact of a mismatch between expectations and experiences. BMC Pregnancy Childbirth. 2021;21(1):475. 34215219 10.1186/s12884-021-03898-z PMC8252193

[7] Preis H, Lobel M, Benyamini Y. Between Expectancy and Experience: Testing a Model of Childbirth Satisfaction. Psychol Women Q. 2018;43(1):105–17.

[8] Garthus-Niegel S, von Soest T, Knoph C, Simonsen TB, Torgersen L, Eberhard-Gran M. The influence of women’s preferences and actual mode of delivery on post-traumatic stress symptoms following childbirth: a population-based, longitudinal study. BMC Pregnancy Childbirth. 2014;14:191. 24898436 10.1186/1471-2393-14-191 PMC4053555

[9] Falk M, Nelson M, Blomberg M. The impact of obstetric interventions and complications on women’s satisfaction with childbirth a population based cohort study including 16,000 women. BMC Pregnancy Childbirth. 2019;19(1):494. 31829151 10.1186/s12884-019-2633-8 PMC6907327

[10] Soderquist J, Wijma K, Wijma B. Traumatic Stress after Childbirth: The Role of Obstetric Variables. J Psychosom Obstet Gynecol. 2002;23(1):31–9. 10.3109/01674820209093413 12061035

[11] Mei JY, Afshar Y, Gregory KD, Kilpatrick SJ, Esakoff TF. Birth Plans: What Matters for Birth Experience Satisfaction. Birth. 2016;43(2):144–50. 26915304 10.1111/birt.12226

[12] National Institute for Health and Care Excellence. NICE guideline [NG201]: Antenatal care. 2021.

[13] Health and Safety Executive. National Standards for Antenatal Education in Ireland. 2020.

[14] Gagnon AJ, Sandall J. Individual or group antenatal education for childbirth or parenthood, or both. Cochrane Database Syst Rev. 2007;2007(3):Cd002869. 17636711 10.1002/14651858.CD002869.pub2 PMC6999801

[15] Yuill C, McCourt C, Cheyne H, Leister N. Women’s experiences of decision-making and informed choice about pregnancy and birth care: a systematic review and meta-synthesis of qualitative research. BMC Pregnancy Childbirth. 2020;20(1):343. 32517734 10.1186/s12884-020-03023-6 PMC7285707

[16] Bailey JM, Crane P, Nugent CE. Childbirth Education and Birth Plans. Obstet Gynecol Clin N Am. 2008;35(3):497–509. 10.1016/j.ogc.2008.04.005 18760232

[17] Rydahl E, Declercq E, Juhl M, Maimburg RD. Cesarean section on a rise-Does advanced maternal age explain the increase? A population register-based study. PLoS ONE. 2019;14(1):e0210655. 30677047 10.1371/journal.pone.0210655 PMC6345458

[18] Chu SY, Kim SY, Schmid CH, Dietz PM, Callaghan WM, Lau J, et al. Maternal obesity and risk of cesarean delivery: a meta-analysis. Obes Rev. 2007;8(5):385–94. 17716296 10.1111/j.1467-789X.2007.00397.x

[19] Sharpe AN, Waring GJ, Rees J, McGarry K, Hinshaw K. Caesarean section at maternal request–the differing views of patients and healthcare professionals: a questionnaire based study. Eur J Obstet Gynecol Reprod Biol. 2015;192:54–60. 26151240 10.1016/j.ejogrb.2015.06.014

[20] National Institute for Health and Care Excellence. Inducing labour (NG207). 2021. 35438865

[21] Wise J. Alarming global rise in caesarean births, figures show. BMJ. 2018;363:k4319. 30314964 10.1136/bmj.k4319

[22] NHS Digital, Maternity Statistics NHS. England London2021 [ https://digital.nhs.uk/data-and-information/publications/statistical/nhs-maternity-statistics/2020-21

[23] Healthcare Quality Improvement Partnership. National Maternity and Perinatal Audit: Clinical report 2017- revised version. 2017.

[24] NHS Digital. NHS Maternity Statistics, England, 2022-23. 2024.

[25] Deshpande S, Kallioinen M, Harding K. Routine antenatal care for women and their babies: summary of NICE guidance. BMJ. 2021;375:n2484. 34716150 10.1136/bmj.n2484

[26] Kitzinger J. Qualitative research. Introducing focus groups. BMJ. 1995;311(7000):299–302. 7633241 10.1136/bmj.311.7000.299 PMC2550365

[27] Ivanoff SD, Hultberg J. Understanding the multiple realities of everyday life: Basic assumptions in focus-group methodology. Scand J Occup Ther. 2006;13(2):125–32. 16856469 10.1080/11038120600691082

[28] Krueger RA. Focus Groups: A Practical Guide for Applied Research. SAGE; 2014.

[29] Braun V, Clarke V. Using thematic analysis in psychology. Qualitative Res Psychol. 2006;3(2):77–101.

[30] Downe S, McCormick C, Beech BL. Labour interventions associated with normal birth. Br J Midwifery. 2001;9(10):602–6.

[31] Freeman L. Confronting Diminished Epistemic Privilege and Epistemic Injustice in Pregnancy by Challenging a Panoptics of the Womb. J Med Philosophy: Forum Bioeth Philos Med. 2014;40(1):44–68. 10.1093/jmp/jhu046 25503792

[32] Supporting Healthy and Normal Physiologic Childbirth. A Consensus Statement by ACNM, MANA, and NACPM. J Perinat Educ. 2013;22(1):14–8. 24381472 10.1891/1058-1243.22.1.14 PMC3647729

[33] Romano AM, Lothian JA. Promoting, Protecting, and Supporting Normal Birth: A Look at the Evidence. J Obstetric Gynecologic Neonatal Nurs. 2008;37(1):94–105. 10.1111/j.1552-6909.2007.00210.x 18226163

[34] Shub A, Williamson K, Saunders L, McCarthy EA. Do primigravidae and their carers have a realistic expectation of uncomplicated labour and delivery? a survey of primigravidae in late pregnancy, obstetric staff and medical students. Aust N Z J Obstet Gynaecol. 2012;52(1):73–7. 22221113 10.1111/j.1479-828X.2011.01396.x

[35] Hardman K, Davies A, Demetri A, Clayton G, Bakhbakhi D, Birchenall K, et al. Maternity healthcare professionals’ experiences of supporting women in decision-making for labour and birth: a qualitative study. BMJ Open. 2024;14(4):e080961. 38684269 10.1136/bmjopen-2023-080961 PMC11057275

[36] Downe S, Finlayson K, Oladapo OT, Bonet M, Gülmezoglu AM. Correction: What matters to women during childbirth: A systematic qualitative review. PLoS ONE. 2018;13(5):e0197791. 29664907 10.1371/journal.pone.0194906 PMC5903648

[37] Townsend M, Brasell AK, Aafi M, Grenyer BFS. Childbirth satisfaction and perceptions of control: postnatal psychological implications. Br J Midwifery. 2020;28:225–33.

[38] NHS England. Choice and Personalised Care in Maternity, Services. NHS England; 2022 [ https://www.england.nhs.uk/mat-transformation/choice-and-personalisation/

[39] Olza I, Leahy-Warren P, Benyamini Y, Kazmierczak M, Karlsdottir SI, Spyridou A, et al. Women’s psychological experiences of physiological childbirth: a meta-synthesis. BMJ Open. 2018;8(10):e020347. 30341110 10.1136/bmjopen-2017-020347 PMC6196808

[40] Smith J, Plaat F, Fisk NM. The natural caesarean: a woman-centred technique. BJOG. 2008;115(8):1037–42. discussion 42. 18651885 10.1111/j.1471-0528.2008.01777.x PMC2613254

[41] Sandall J, Soltani H, Gates S, Shennan A, Devane D. Midwife-led continuity models versus other models of care for childbearing women. Cochrane Database Syst Rev. 2016;4(4):Cd004667. 27121907 10.1002/14651858.CD004667.pub5 PMC8663203

[42] Perriman N, Davis DL, Ferguson S. What women value in the midwifery continuity of care model: A systematic review with meta-synthesis. Midwifery. 2018;62:220–9. 29723790 10.1016/j.midw.2018.04.011

[43] NHS. Episiotomy and perineal tears: NHS. 2020 [ https://www.nhs.uk/pregnancy/labour-and-birth/what-happens/episiotomy-and-perineal-tears/

[44] Smith LA, Price N, Simonite V, Burns EE. Incidence of and risk factors for perineal trauma: a prospective observational study. BMC Pregnancy Childbirth. 2013;13:59. 23497085 10.1186/1471-2393-13-59 PMC3599825

[45] Merriel A, Toolan M, Lynch M, Clayton G, Demetri A, Willis L et al. Codesign and refinement of an optimised antenatal education session to better inform women and prepare them for labour and birth. BMJ Open Qual. 2024;13(2). 10.1136/bmjoq-2023-002731 PMC11168157 38858078

[46] Waddell A, Spassova G, Sampson L, Jungbluth L, Dam J, Bragge P. Co-designing a theory-informed intervention to increase shared decision-making in maternity care. Health Res Policy Syst. 2023;21(1):15. 36721156 10.1186/s12961-023-00959-x PMC9888748

[47] Lovell H, Silverio SA, Story L, Skelton E, Matthew J. Factors which influence ethnic minority women’s participation in maternity research: A systematic review of quantitative and qualitative studies. PLoS ONE. 2023;18(2):e0282088. 36827386 10.1371/journal.pone.0282088 PMC9956875

[48] World Medical Association. WMA Declaration of Helsinki – Ethical Principles for Medical Research Involving Human Participants. 2013. 10.1001/jama.2024.21972 39425955

