# Supplementary material for: A qualitative exploration of women’s expectations of birth and knowledge of birth interventions following antenatal education
Source: BMC Pregnancy Childbirth. 2024 Dec 28;24:875. doi: 10.1186/s12884-024-07066-x (PMC11682617; doi:10.1186/s12884-024-07066-x)
Supplement: Supplementary file 1 — Supplementary Material 1 [file 12884_2024_7066_MOESM1_ESM.docx]

**Supplementary file 1:** Sociodemographic characteristics by focus group

| **Group number/ N** | **FG1 (n=6)** | **FG2 (n=4)** | **FG3 (n=6)** | **FG4 (n=7)** | **FG5 (n=8)** | **FG6 (n=8)** | **FG7 (n=7)** |
| --- | --- | --- | --- | --- | --- | --- | --- |
| **Mean age (years)** | 32.17 | 33.25 | 33.83 | 33.29 | 34.43 | 34.13 | 32.44 |
| **Age Range** | 29-38 | 32-38 | 27-42 | 26-44 | 32-39 | 23-40 | 24-40 |
| **Ethnicity** |  |  |  |  |  |  |  |
| *White British* | 6 | 3 | 6 | 7 | 6 | 6 | 3 |
| *Any Other white* | 0 | 1 | 0 | 0 | 1 | 2 | 1 |
| *Black/Black British/ Asian/ Other minority ethnicity* | 0 | 0 | 0 | 0 | 1 | 0 | 2 |
| *Did not indicate* | 0 | 0 | 0 | 0 | 0 | 0 | 1 |
| **Education** |  |  |  |  |  |  |  |
| *GCSE/A-Levels* | 0 | 0 | 2 | 0 | 0 | 2 | 0 |
| *Degree* | 2 | 3 | 2 | 3 | 5 | 4 | 5 |
| *Post-graduate Degree* | 4 | 1 | 2 | 4 | 3 | 2 | 1 |
| *Did not indicate* | 0 | 0 | 0 | 0 | 0 | 0 | 1 |
| **Invited to NHS Antenatal** |  |  |  |  |  |  |  |
| *Yes* | 5 | 1 | 4 | 6 | 6 | 8 | 6 |
| *No* | 1 | 3 | 2 | 1 | 2 | 0 | 1 |
| **Attended NHS Antenatal** |  |  |  |  |  |  |  |
| *Yes* | 2 | 1 | 3 | 4 | 3 | 5 | 5 |
| *No* | 4 | 3 | 3 | 3 | 5 | 3 | 2 |
| **Attended Private Antenatal** |  |  |  |  |  |  |  |
| *Yes* | 5 | 3 | 4 | 3 | 7 | 3 | 2 |
| *No* | 1 | 1 | 2 | 4 | 1 | 5 | 5 |
| **Attended both NHS & Private** | | |  |  |  |  |  |
| *Yes* | 1 | 0 | 1 | 1 | 3 | 2 | 2 |
| *No* | 5 | 4 | 5 | 6 | 5 | 6 | 5 |
| **Age of baby (weeks)** |  |  |  |  |  |  |  |
| *0-12* | 3 | 0 | 1 | 1 | 1 | 1 | 0 |
| *13-24* | 3 | 2 | 1 | 2 | 2 | 2 | 1 |
| *25+* | 0 | 2 | 4 | 4 | 5 | 5 | 6 |
| **First/ subsequent baby** |  |  |  |  |  |  |  |
| *First* | 5 | 1 | 2 | 5 | 6 | 6 | 5 |
| *Subsequent* | 1 | 2 | 4 | 2 | 2 | 2 | 2 |
| *Previous pregnancy loss* | 0 | 1 | 0 | 0 | 0 | 0 | 0 |
| **Method of delivery** |  |  |  |  |  |  |  |
| *Spontaneous vaginal* | 5 | 3 | 3 | 4 | 5 | 3 | 3 |
| *Instrumental* | 0 | 0 | 0 | 1 | 1 | 0 | 2 |
| *C-section: Emergency* | 0 | 0 | 0 | 1 | 0 | 2 | 2 |
| *C-section: Not specified* | 1 | 1 | 2 | 0 | 1 | 2 | 0 |
| *C-section: Elective* | 0 | 0 | 1 | 1 | 1 | 1 | 0 |
| **Change in location during birth** | | | | | | | |
| *Yes* | 0 | 1 | 0 | 0 | 1 | 2 | 2 |
| *No* | 6 | 3 | 6 | 7 | 7 | 6 | 5 |
| **Any complications in birth** | | | | | | | |
| *Yes* | 2 | 1 | 1 | 5 | 1 | 5 | 4 |
| *No* | 4 | 3 | 5 | 2 | 7 | 3 | 3 |
