# Supplementary material for: A qualitative exploration of women’s expectations of birth and knowledge of birth interventions following antenatal education
Source: BMC Pregnancy Childbirth. 2024 Dec 28;24:875. doi: 10.1186/s12884-024-07066-x (PMC11682617; doi:10.1186/s12884-024-07066-x)
Supplement: Supplementary file 2 — Supplementary Material 2 [file 12884_2024_7066_MOESM2_ESM.docx]

**Supplementary File 2: Additional quotes from theme 1- Education as a source of optimistic beliefs about a ‘natural’ birth**

| **Hierarchy of birth and pain relief**  *Yeah it [pain relief] definitely it did feel hierarchical, it might have been a bit more neutral and a bit more balanced. Yeah to really in preparing the different options and knowing what would be available in such and such a circumstance and then this happened then actually that’s not going to be available. Yeah. (FG1)*  *Probably the absolute ideal scenario but given that doesn’t happen even with the best intentions for a lot of people, then there needs to be a bit more sensitivity towards you can still feel really good about your birth, you can still have a really good birth even if you have to have some medical interventions. This is what they are. This is what it looks like and this is what it’ll be afterwards. It sounds bad but it’s not necessarily that bad. (FG1)*  *…. I agree with the realistic view. Information. Neutral. Signposting. Where to go for it. Whatever happens, it’s okay and there’s a stage beyond the birth, remember even the first couple of hours. As I said, I don’t know if I’d have been that open to it but I think it probably is important to try and encourage people to look beyond the birth. There isn’t a good birth or a bad birth. It’s not that you’re achieving something by having no pain relief and no tearing, it’s that we’re lucky really. Plus, there are things you can do to prepare and this is them. But still there’ll be more surprises. I don’t know, maybe that was already trying to be the message but it wasn’t maybe what I’ve heard. I think this meant others, I’m not sure it’s what some other people heard, so there must be reasons for that. (FG1).* |
| --- |
| **Expecting an uncomplicated birth**  *I think it’s really difficult balance. In one way, it’s good not to make women anxious and they’re trying to say, your body can do this and it’ll all be fine and lovely. I see the benefit of not worrying people, but in another way, it’s a little bit like pulling the wool over your eyes and not just preparing for what actually might happen or making them feel worse about it not going that way.(FG1)*  *Yes the [name] classes, I remember focussing so much on how sort of the lovely ways that you could give birth. So you could have a water birth or a home birth or this or that and I knew from day one I wanted to be in a hospital, surrounded by a team of medical professionals and there kept being comments about, but in a birthing centre you have midwives. I said yes, midwives are incredible, they know what they’re doing but if something goes wrong, I don’t want to be in a birthing centre. So the [name] classes are very tailored for – you can give birth in a field and it will be lovely so they don’t cover a lot of the complications and don’t prepare you. Well yes, they don’t prepare you enough for actually you’re gonna walk into a room, you’re gonna have an amazing midwife who’s gonna press a button if your baby poos when it’s coming out and you’re gonna have a team of doctors. That won’t happen in a field. [laugh] [FG3)*  *I kind of thought mine would be quite straightforward, no induction. I think there wasn’t a lot about… well, I was induced actually due to his size and I didn’t have a lot of information. I was quite shocked when I was told that was the reason I was being induced because I didn’t have a lot of information. (FG6)*  *Literally, I genuinely believed I’d be home the next day, I was like this is it, she’s coming and then it got closer and closer to the weekend and then the weekend came and went and then it was like, oh, yeah. Even if it was just a leaflet on it. I’m sure there is a leaflet on it, but [over talking - 50:39] because I didn’t have a clue. I got induction then, bam, you’re having it, pop, out it comes and although you’d have to work harder to get it out you would have it. But actually it was five days of different things trying to make it come and, actually, when it came to it there were complications and things. (FG7)*  *I felt like there was quite a lot of emphasis on the midwife led stuff and I remember a reasonable amount being dedicated to talking about the aromatherapy and all of these things, which I don’t know if it was more in hindsight or even a little bit at the time, I almost felt a little bit irritated because that’s assuming that the rooms are available and there’s less midwife led rooms than there are medical and [unclear]. It’s nice if you can have those things but I felt like the balance was more on that when actually you’re probably less likely to end up in a bath with aromatherapy. Even though that would be really nice, it’s almost setting yourself up for a fall if you then don’t – I don’t know it felt like more time was dedicated than that, than complications or… (FG1)*  *Yeah, it’s just a real emphasis on how nice and wonderful all the lovely midwife led rooms are. Loads about how great the facilities are in the midwife led rooms. I just felt like there was a lot of emphasis on that rather than other things. They do sound nice but it’s just like bad luck if you don’t get it.(FG1)*  *And I think there's a difference between positively talking about birth as a natural thing, which many women have done before and everyone will be able to do. And almost like talking about this in a way that doesn't make it seem like your body has failed if one of these things happens to you, because I think, I think that's almost like where this funny sort of thin line is. All births are good births because even, even if the absolute worst happens, that it's still like your body, you know, it's not that you failed. (FG2)* |
| **Planning, control and choice**  *I think before I went into the antenatal education, I think I was like I had no control over what birth I had and that it’s kind of up to your body what happens and then I was quite surprised by the antenatal education just pushing the idea that you’d have lots of control over what happens in your birth. I think sort of can guide it a bit, but ultimately what your body does is what your body does and that was a surprise for me. (FG1)* |
| **Keeping an open mind and preparing to be flexible** *We were sort of encouraged to hope that everything would go straightforward but we went through a role play of what happens when you go to theatre and what different complications might happen and like why you might want an epidural, why, why an intervention might happen. (FG4)* *I think the classes actually prepared me in some way for my first pregnancy because it opened my eyes to the fact that the birth could go a number of different ways and I guess I bought into the fact it could be any one of them. I didn’t really hang my hopes on one or the other. (FG6)*  *I think that was with positive birth where they take you through birth planning and they try and get you into the head space where you consider what would happen if things change. That’s right down to if you have a caesarean you might want the electrodes on your back so that you can have the child put on your stomach kind of thing, so quite good detail about that. (FG5)* |
| **An expectations-outcome mismatch**  *It’s really hard when you’re in a situation where everything is done by a textbook, and birthing you definitely don’t go by text book so when they say you have to wait until your contractions are X minutes apart that’s fine as long as your resp rate is like that, but in reality some of them are quicker. (FG4)*  *I thought I was gonna go in, have a baby and come out within two hours. (FG7)But every single stage was a shock ’cause I didn’t know what to expect and every single stage was extremely painful for me and my body and my baby. I think that, yeah, that was a real eye opener because I didn’t expect it.(FG7)* |
