# Supplementary material for: A qualitative exploration of women’s expectations of birth and knowledge of birth interventions following antenatal education
Source: BMC Pregnancy Childbirth. 2024 Dec 28;24:875. doi: 10.1186/s12884-024-07066-x (PMC11682617; doi:10.1186/s12884-024-07066-x)
Supplement: Supplementary file 3 — Supplementary Material 3 [file 12884_2024_7066_MOESM3_ESM.docx]

**Supplementary file 3: Additional quotes from theme 2- *Knowledge of interventions/complications as a means of preparing for making choices and coping with labour and birth experiences.***

| ***Information can enhance wellbeing but should be managed prevent anxiety*** *I don’t think induction is covered particularly well in NHS class. (FG4)**We did a whole session in [private provider] on induction and sort of that kind of thing as well. (FG4)* *I felt like I kind of roughly knew that the rates in terms of your reasonable chance of intervention but I feel like the emphasis is that it’s not that likely to happen. I feel like if I hadn’t known that, the message is like you should be fine. That you’re less likely to have any of these things so try not to worry too much about them rather than they’re actually a bit more common than the perception that was given. (FG1)* *I’d say the same in regards to the induction process as well [baby crying 16:18] but I know that a lot of people can take days and days and when it came to my induction and they were talking to me about …. I felt like I didn’t know a lot about that prior. I kind of knew what kind of things they do. (FG6)* *They did talk about inductions. We had a bit of a thing about these are all the different stages. This is what they’ll do first and this how long we have to wait and they did all the stages for what to expect in an induction. (FG1)*  *They did also have a little bit about caesarean as well and a very brief nod to the fact that we might have an emergency caesarean. I remember they did talk about that. (FG1)*  *You'd be just be a bit more prepared in a way. But I guess it depends on the individual but some people might get worried, but I'd rather just be told, this is what could happen, this is what might happen. (FG2)*  *Interesting because I think for me it doesn’t make a difference if once I’m pregnant, whatever demographic I fell into I’d want to know the statistics. For me I think if I was – I don’t know. I feel like whatever, if it’s about overweight or your age or your ethnicity because there are differences again, I’d just rather have the information. But I don’t know if that’s just me or whether that’s… (FG2)*  *I think I, again I’m just talking personally, but I just think having a chart like this would be really useful to tell people what's going on right now and normalizing it. And you know that these things do happen. Yeah to be prepared. (FG2)*  *I think the elective caesareans are interesting as well. It’s amazing how many people say, how many people say that they oh I don't know if I want, I chose to have one, like it's a bad thing. But I had a friend, she went through the long labour, had the forceps, that had to have the emergency caesarean. So second time around, she had an elective caesarean and she was covering up it up like it was a bad thing. (FG2)*  *I feel like that the undercurrent almost of glossing over some of these more difficult aspects of the complications that can arise is that there's, there's, uh, a feeling that you failed, if one of these things happens to you instead of talking about them as the less likely than an assisted delivery situation. I mean, this, this tells you that most births are spontaneous vaginal deliveries. Now, for me looking at that, I'm like, wow, that's amazing. And actually, if you, if you take the 10% of electives caesareans out of that too, it’s even more of a percentage that actually it just works. And, but if it doesn't work, these are the things we're going to do. These are the decisions you might have to make um, and actually it's a very matter of fact thing. It's not scary. It's not an overload of information necessarily, but I think something like this, this is actually just a chart like this is really helpful because it could in a class, like a short class, give people a chance to say, to ask a few questions and then be pointed in with where to get further information. (FG2)*  *I think I had a very difficult labour, I didn’t know it could be [inaudible - 15:35], because it’s like the, like you said, the perfect scenario [baby noise - 15:38], they’re not just trying to scare you, but the other options that were available. So if you get pre-eclampsia you might have to stay in for a week ’cause [inaudible - 15:47]. These are things that might count as an emergency. ’Cause she was already trying to push me to [baby noise - 15:54], it’s almost just like there could never be anything wrong, that whole – it just…(FG7)*  *What I briefly remember is going through everything and then them saying obviously if you’re induced none of that applies but then not really saying what did apply and people were just [unclear 23:51]. We did discuss how many women were induced like in the local area but yeah it obviously puts a whole different slant on the whole process. (FG4)* |
| --- |
| ***Understanding why interventions and complications happen can help women prepare***  *I thought inductions were increasing because of guidelines. (FG1)*  *Do any of you have any ideas about what factors mean that you end up with one of these interventions both during the birth and maybe?*  *P: Baby weight.*  *I: Okay so baby weight.*  *P: Gestational diabetes but then that links to baby weight isn’t it so.*  *I: Okay so they’re connected, yes.*  *P: Long labours.*  *I: Long labour, yes.*  *P: Whether you’re induced or not.*  *P: Baby in the wrong position. (FG2)*  *I had a kidney problem so I had to be induced at 37 weeks. (FG7)*  *You see I had to have an induction because I had a 36 week scan booked in to check whether he’d turned or not and they found no waters left so he came out then. I had to have a 12 hour pessary not a 24 hour pessary and if I hadn’t gone into labour within those 12 hours, I was in for a C section. They gave him 12 hours to start trying to come out and that was it, so I was very lucky that he did. (FG3)*  *I don’t know, yes. People I know who are older know. (FG1)*  *Don’t think I could say a percentage but the research suggested the numbers are increasing recently, especially with inductions but I can’t remember…(FG1)*  *I don’t remember there being much on the C-section in the NHS one. I feel like the emphasis was more like you can assume that it won’t be very likely. I was like even if the stats were 25 percent, that’s quite high. I feel like they should have given a little more time to it. (FG1)*  *I have been told that caesareans was something like 30% at [hospital trust] which now I think seems very low compared to the number of women that I know that had caesareans. And I don't know whether that is a statistic that has been grouped together with mothers who like second time moms, third time moms. Because I think that the statistic would be very different if it were just first time moms. (FG2)*  *Like she has very big babies and very small hips and my mum was the same, you know, really struggled with labour and she had…(FG2)* |
| ***What they don’t tell you about- impact of birth on the body***  *Only a little bit. But again, like, breastfeeding tearing, those two things weren’t really talked about much and those my worst things I was prepared for… (FG2)*  *I, I recall that was not something I thought would happen until I didn't realise third degree tears, you know what it meant and it meant stitches in theatre. (FG2)*  *but it’s just that thing, they say to you about episiotomy, you just don’t know how painful that is. (FG7)*  *Pelvic floor stuff, I don’t remember that once, so in my head I don’t think I realised the importance of doing it. Literally you have no – if you’ve got a cold and cough when you’re giving birth you’ve got no control [inaudible - 26:17], it’s like you are just gonna wee. And for someone to be a bit more, not to scare you, ’cause I know you probably only listen when it’s happening to you, but to be a bit more like it’s really important. (FG7)*  *We did a whole bit on perineal massaging as well [in private class]. (FG4)*  *So what to expect from your body, what it will look like, what it will feel like and dealing with discomfort and yes. (FG3)*  *We briefly covered Episiotomy and sort of tears, I guess they are all the same…(FG4)*  *We did talk about it in the NHS and we talked about the different degrees of tear and the thing I remember coming away with was I do not want my bottom to tear! I think that was the message that they drummed into me! I remember my mum saying to me ‘Oh they make a little cut’ and I was like [inaudible 38:24] and then I came away from that thinking actually an episiotomy might be quite a good thing to have! (FG5)*  *We talked about perineal massage but the NHS class said don’t bother. (FG5)*  *We talked about it in the other groups - not the NHS group. They [baby noise 39:33] episiotomy, they were also more reserved about it in the positive birth groups where I think there’s some research – there’s probably other research that says the opposite – that suggests that it’s not always the best course and women can have a lot of side effects from the episiotomy so there’s a sort of debate going on that they were talking about in those classes. (FG5)*  *I had an episiotomy and I didn’t know how to deal with that at all. I kind of knew why they did it but I didn’t really know then how to deal with everything afterwards and… (FG6)*  *Yeah. I think more of an idea of how long it will take to recover as well because after two days I was asking the midwife why I wasn’t recovering and she was like ‘well, you gave birth two days ago so… (FG6)*  *Yeah, and I was told a lot because I was young I would heal brilliantly and perfectly but actually I found it a lot harder than the actual giving birth part. (FG6)* |
